# Development of Low-Cost IoT System for Monitoring Piezometric Level and Temperature of Groundwater

**DOI:** 10.3390/s23239364

**Published:** 2023-11-23

**Authors:** Mauro Espinoza Ortiz, Juan Pablo Apún Molina, Salvador Isidro Belmonte Jiménez, Jaime Herrera Barrientos, Héctor José Peinado Guevara, Apolinar Santamaria Miranda

**Affiliations:** 1 Programa de Doctorado en Red en Ciencias en Conservación del Patrimonio Paisajístico, CIIDIR Unidad Sinaloa, Instituto Politécnico Nacional, Guasave C.P. 81100, Sinaloa, Mexico; mespinozao1700@alumno.ipn.mx; 2 CIIDIR Unidad Sinaloa, Instituto Politécnico Nacional, Guasave C.P. 81100, Sinaloa, Mexico; asantama@ipn.mx; 3 CIIDIR Unidad Oaxaca, Instituto Politécnico Nacional, Santa Cruz Xoxocotlán C.P. 71230, Oaxaca, Mexico; 4 Centro de Investigación Científica y de Educación Superior de Ensenada, Baja California (CICESE), Ensenada C.P. 22860, Baja California, Mexico; jherrera@cicese.mx; 5 Escuela de Ciencias Económicas y Administrativas, Universidad Autónoma de Sinaloa, Guasave C.P. 81100, Sinaloa, Mexico; hpeinado75@hotmail.com

**Keywords:** aquifer, piezometric level, well monitoring, wireless sensors

## Abstract

Rural communities in Mexico and other countries with limited economic resources require a low-cost measurement system for the piezometric level and temperature of groundwater for their sustainable management, since anthropogenic action (pumping extractions), natural recharge and climate change phenomena affect the behavior of piezometric levels in the aquifer and its sustainability is at risk. Decrease in the piezometric level under a balanced level promotes salt intrusion from ocean water to the aquifer, salinizing and deteriorating the water quality for agriculture and other activities; and a decrease in water level under the pumps or well drilling depth could deprive communities of water. Water temperature monitoring is essential to determine electric conductivity and dissolved salt content in groundwater. Using IoT technology, a device was developed that monitors both variables inside the well, and the ambient temperature and atmospheric pressure outside the well. The measurements are made in real time, with sampling every second and sending data to a dedicated server every 15 min so that the visualization can be accessed through a device with Internet access. The time series of the variables measured inside and outside the well were obtained over a period of three months in the rural community of Agua Blanca, Guasave, Sinaloa, Mexico. Through these records, a progressive temporary drawdown of the piezometric level is observed, as well as the frequency of pumping. This low-cost IoT system shows potential use in hydrological processes of interest such as the separation of regional and local flow, drawdown rates and recognition of geohydrological parameters.

## 1. Introduction

Groundwater is a vital resource, essential for agriculture, domestic use, industry, and environment. Rapid economic growth, population increase, urbanization, and the continued expansion of human development have aggravated water scarcity in many basins [1]. The management of groundwater resources is an important issue, especially regarding agricultural potential [2]. However, due to the lack of public policy and supervisory measures for its use, overexploitation in some aquifers has been extensive, altering flow regimes and thus becoming a threat to socioeconomic development and ecological health [3].

Thus, piezometric level and temperature monitoring are necessary in sustainable management to prevent negative impacts. Piezometric monitoring allows one to take actions to avoid salt intrusion into the aquifer [4,5], caused by a hydrostatic imbalance as a result of different densities between saltwater from the Pacific Ocean and conditioned diminution by pumping continental freshwater; to prevent water deprivation in certain communities and well weakening due to decrease in piezometric level under pumps or well drilling depth; or identify water level increase in topographic depression that generates flood zones [6]. Temperature monitoring enables one to determine electric conductivity in groundwater, which is correlated with water quality by dissolved salt content and piezometric level [7].

In addition, monitoring the piezometric level is required to design recharge strategies for the aquifer [8], to prevent the levels from dropping, causing sinking and loss in the storage capacity of the aquifer, and to know the flow direction of groundwater and possible contamination risks [9].

The global extraction of groundwater has increased considerably in recent years, generalizing the overexploitation of aquifers and resulting in a decrease in the piezometric level, which causes greater energy consumption by increasing the pumping head [10]. The impact of extractions and recharges are not well known until months or years after the event, which makes sustainable groundwater management difficult and motivates modernization with real-time technology [11]. To achieve the correct management of water resources, constant monitoring of water parameters is necessary [12], as the continuous measurement of the piezometric level in groundwater is one of the main tools for any study on the effective characterization of an aquifer and flow models [13].

Continuous groundwater monitoring is mainly used to estimate changes in aquifer storage [14], calibrate groundwater flow models [15,16] and provide updated information to agencies responsible for implementing water resource management legislation [17].

Well monitoring provides direct in situ measurements of the depth below the natural ground surface at which water is found; this technique is used in most aquifers from measurement networks [18,19]. The area of study and the temporal frequency of measurements are usually different and vary in terms of cost, scalability, and ability to answer scientific and management questions [13]. Otherwise, there are commercial automated systems for monitoring groundwater that remain fixed in the well for months or years, collecting data with a specific temporal monitoring frequency and recording data for later analysis; however, they tend to be unaffordable due to their high cost to be applied in an aquifer monitoring network [11].

In recent years, with the development of technology, the reduction of costs in hardware, expansion in software supported on the open-source community, the development of real-time monitoring systems by researchers and engineers has increased [20,21]. These systems are low-cost wireless sensors, built from affordable electronic components, enabled by telemetry to provide real-time data [22].

Systems connected with the Internet of Things (IoT) offer opportunities in multiple areas of research to solve new problems [23,24]. Low-cost wireless sensors have been widely developed in different areas, to measure the value of particles suspended in the air [25], monitoring of geothermal systems [21], climate stations with artificial intelligence for smart farms [26], radon gas monitoring system for smart homes [27], hydrological monitoring of landslide-prone areas [28], and for the study of fish behavior [29]. Previous works agree on the need to develop low-cost sensors since their application on large scales significantly reduces the costs and times to generate information [11].

The objective of this work is to generate a low-cost system designed for rural Mexican communities based on IoT technology for real-time groundwater monitoring. This system has advantages such as not requiring a specific calibration control when it is installed, unlike others on the market, and data are available for visualization through open source libraries.

## 2. Materials and Methods

The prototype measures in situ and in real time the piezometric level and temperature inside the well, in the saturated zone, as well as the ambient temperature and atmospheric pressure outside the well. The data obtained are sent to a web page for subsequent analysis or displayed in graphic form or tables. The prototype components are described in the following sections.

### 2.1. Component Overview

The depth of groundwater is obtained using a high-performance pressure transducer from the DFRobot brand (KIT0139; [30]). The pressure transducer is encapsulated in an industrial stainless-steel probe. When the probe is immersed in the saturated zone, it will be exposed to the pressure by the liquid column. This pressure is converted into a current signal (4–20 ma) that is converted to analog voltage, compatible with most current microcontrollers. An ADS1115 analog-to-digital converter with 16-bit resolution is used, which is connected to the microcontroller through the I2C protocol. The ESP8266 microcontroller is used, integrated with a 2.4 Ghz WiFi antenna ideal for IoT tasks, powered with 5 V via micro-USB.

**Figure 1:**
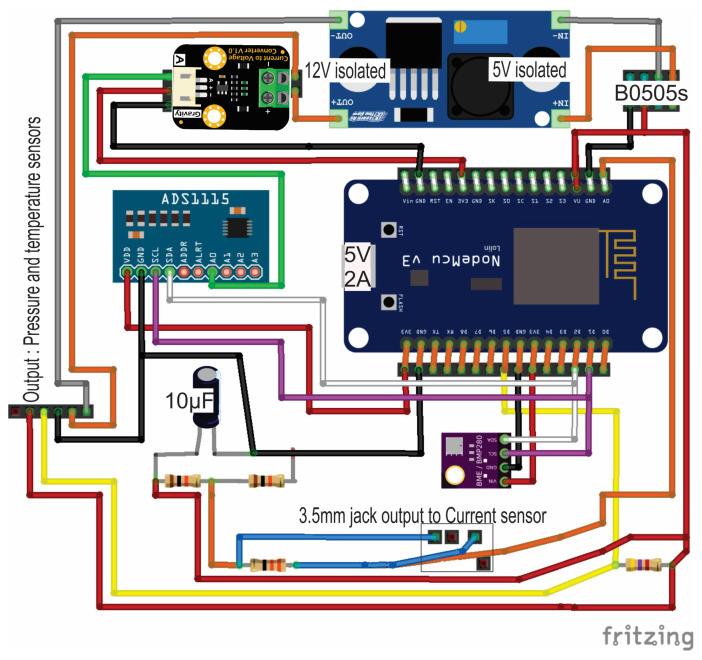
Electronic diagram of the connection between components used in the prototype.

Due to the fact that the pressure sensor has an operating range of 12 to 36 volts, an isolated electrical connection was made to reduce electrical noise interference with an isolated DC-DC converter (B0505S-2W) Figure 1. The ESP8266 microcontroller was powered with a conventional alternating current regulator at 5 V, 2 A direct current (DC) as the main power source and connected to an isolated DC-DC converter to power the DC-DC booster (Xl6009e1). This has an approximate efficiency of 94% and, in this way, it regulates the 12 V required by the pressure sensor.

In addition to the pressure sensor, temperature and atmospheric pressure sensors were placed. The temperature sensor uses the waterproof Ds18b20 chip and is submerged with the pressure transducer. The piezo-resistive sensor (BME-280) was used with an operating voltage of 3.3 V that measures ambient temperature, humidity, and atmospheric pressure.

For detecting and recording the water extraction pump operation in a well, a non-invasive current sensor (SCT-013) was added. This record is important to know the consumptive uses of groundwater, its frequency of use.

Figure 1 shows the configuration of the PCB (printed circuit board). This figure was created with Fritzing software, version 0.9.3 (open source).

**Figure 2:**
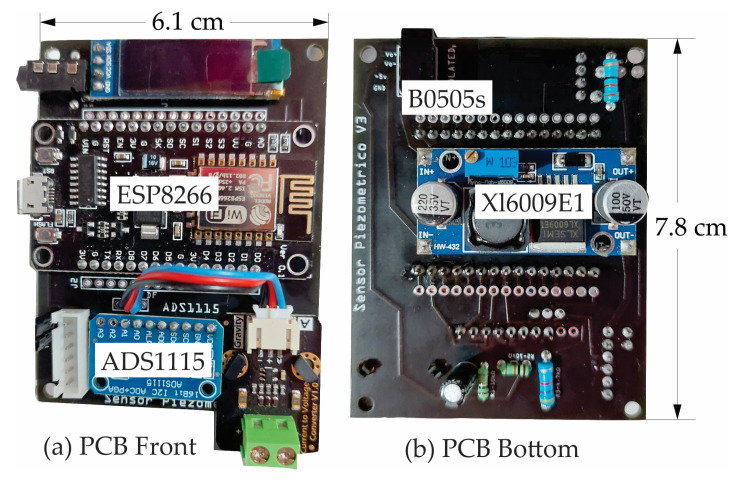
Prototype printed circuit board.

The circuit was designed in 6.1 × 7.8 cm size (Figure 2) and placed inside a cylinder 4 inches in diameter and 8 inches long that acts as the axis of a reel on which the cable that connects the sensors for measuring the variables: piezometric level, temperature, and atmospheric pressure.

### 2.2. Prototype Operation

The code that allows reading the sensor data from the ESP8266 module was written with the Arduino IDE programmer. Using the WiFiManager library [31], through a mobile device via WiFi, it connects to the microcontroller to configure Internet network credential and initial values from well, this data are stored in the internal memory of the ESP8266 module.

The PubSubClient library [32] is used to publish the data of the measured variables on a server, while the ESP8266 module is configured to send the information on a server (BROKER), using an address compatible with the protocol MQTT (Message Query Telemetry Transfer) on a public or personal server. The ESP8266 microcontroller processes the information from the sensors and publishes it to a server.

### 2.3. Prototype Installation at Monitoring Site

**Figure 3:**
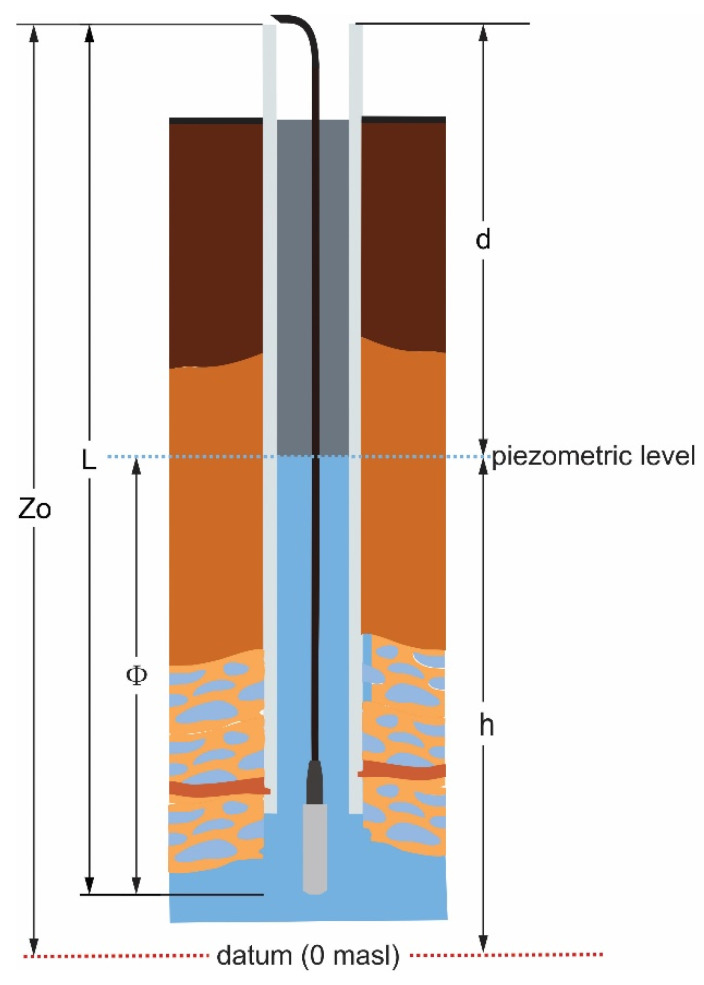
Diagram of prototype operation.

The prototype is configured with the site information: name of the site, elevation of a reference point on the ground surface (Zo) or well curb, and the total length of cable introduced into the well (L), measured from the Zo point (Figure 3). These variables are added in situ to the code via a mobile device connected to the ESP8266 through the WiFiManager library.

In this way, the elevation of the groundwater level (h) of the groundwater can be obtained using the following equation.

h = Zo − L + Φ(1)

h = Zo − d(2)

where:

Zo = Reference point on the ground surface or well curb (masl).

d = Depth of the piezometric level (m).

L = Length of cable introduced into the well measured from the reference point (m).

Φ = Height of the water column from the sensor to the water level (m).

h = Height of the piezometric level (masl).

The ESP8266 module was configured to publish data to a server using the MQTT protocol every 15 min to prevent saturating with redundant data. The publication of the recorded data was also established once the elevation of the piezometric level (h) undergoes a variation (increase or decrease) of 2 cm with respect to the initial value of the window. This is to record changes produced by factors external to the well and internal ones due to extraction by pumping. So, during the 15 min window, the sensor samples the piezometric level every second, the last reading is recorded at the limit of the window and then it is sent to the server. But, if the data change with respect to the first reading in the window by two centimeters or more, the new reading is recorded and immediately sent to the server without waiting for the window to end.

### 2.4. Communication Diagram

Given the restrictions on the number of devices that can be connected to a commercial server consulted for this work, the amount of data that can be sent per unit of time and costs associated with these, a server was implemented using a Raspberry pi 4 B with a Broadcom BCM2711 processor, Quad core Cortex-A72 @1.8 Ghz, 8 GB of RAM, with the Raspberry Pi OS (64-bit) operating system, and Apache Web server (open source) was installed as a dedicated HTTP web server [33]. The MQTT protocol Eclipse Mosquitto [34] was installed as a broker for receiving data sent by the prototypes with the ESP8266.

The MariaDB library [35] was used as relational database to manage, store and consult data received by various devices. Using the paho.mqtt.client library [36], a connection is established from the MQTT client to the Raspberry server and the data are received and stored in the tables.

To consult information from the databases, PHP, HTML and JavaScript programming languages were used. With the Chart.js library [37], the variable data are displayed in graphs and/or tables. The server uses the Leaflet library [38] to generate interactive maps and show the position of connected devices.

Accessibility to the server from any device connected to the Internet was configured to the dynamic domain name system (DDNS) with the NO-IP domain provider responsible of directing device requests to the Raspberry server with the free domain http://piezometriaguasave.ddns.net (accessed on 1 June 2023).

**Figure 4:**
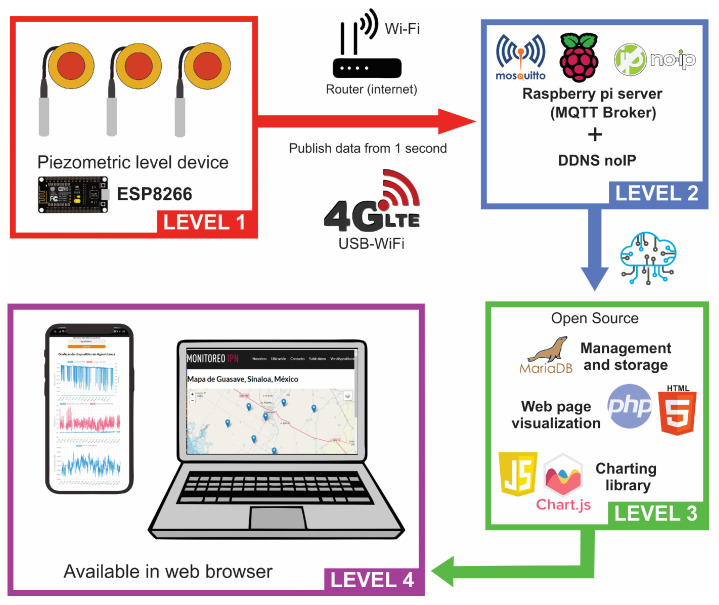
IoT schematic of the monitoring system; data visualization at level 4 available in http://piezometriaguasave.ddns.net (accessed on 1 June 2023).

The communication operation diagram is shown in Figure 4. Level 1 represents the sensors connected to the ESP8266 module that send the variables to the Raspberry Broker through the MQTT protocol and Internet connection. At level 2, the Broker receives it, records the identifier name of each ESP8266 and the date-time of reception using Python script designed to give access to database. Subsequently, at level 3, the variables are stored in the database through the use of MariaDB, visualized as a web page with PHP and HTML and the data are graphed with Chart.js—all of these are open-source libraries. The query is made at level 4 by visiting the domain from any PC or device web browser with Internet access; the Raspberry web server shows graphs of the stored data.

### 2.5. Design, Construction, Assembly and Operation of the Prototype

The main tool used was computer-aided design (CAD) in SOLIDWORKS 2019 software; this software allowed us to design, analyze and visualize a 3D model.

**Figure 5:**
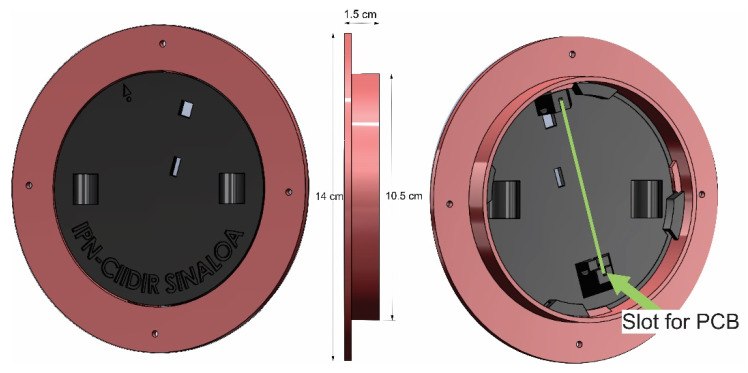
View of designed structure that assembles in 4″ PVC tube and assembly position of the PCB.

**Figure 6:**
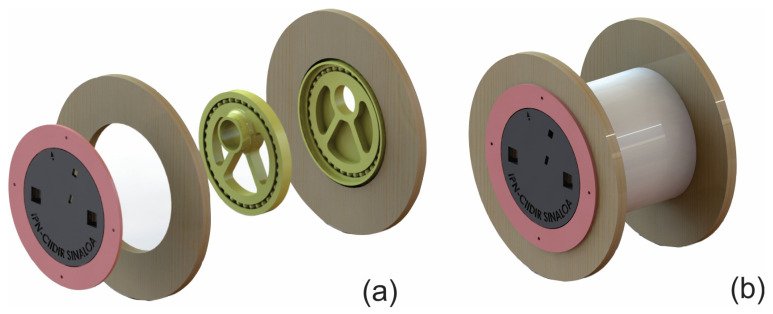
Final CAD design of the piezometric sensor prototype: (**a**) exploded view, (**b**) general view of the design.

**Figure 7:**
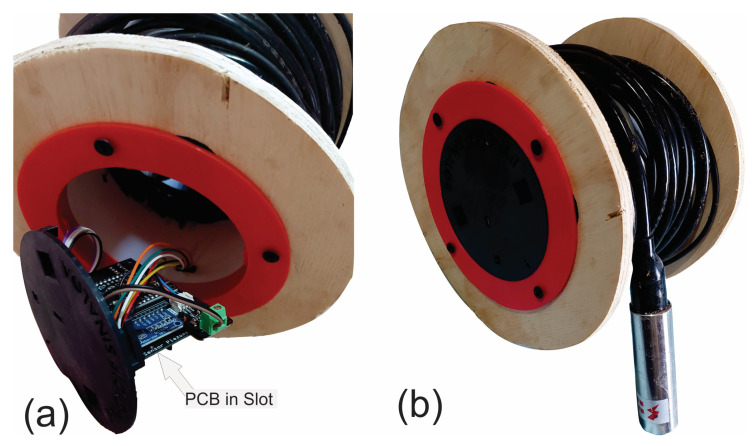
Final view of the prototype. (**a**) View of the structure that holds the PCB to the reel. (**b**) Developed piezometric level.

A reel was used so the electronic components can be inside a 4″ diameter and 8-inch-long PVC pipe. Figure 5 shows the design for assembling the PCB that allows secure assembly on the PVC pipe. Two bearings were placed (Figure 6a): one exterior that allows the placement of pine wood (Figure 7) and another that goes inside to support the reel and facilitate the cable winding. All designs were printed on a 3D plastic printer (Creality Ender 3) using PLA-type plastic filament.

The reel structure was designed with the capacity to wind up to 30 m of cable. The device was installed by an operator who traveled to the site; there, it was configured to work correctly. It is not necessary for the well owners to have technical knowledge about maintenance and/or repair; it is only necessary to be aware of the existence of a monitoring device and its importance for household and community water use. So, to reinforce awareness, we informed the well owners about the false belief that groundwater is unlimited and that it is only produced for human needs, also explaining that, in case of poor quality or lack of water, the state will solve the problem but transfer the costs mainly to them, making their economic and social situation precarious.

The device can be submerged for long periods (months) and function correctly; however, the possible failures that could occur are considered from the server. If the device stops transmitting, it is assumed that there is a problem and the operator, after calling the well owners, tries to solve the problem with his support. If this cannot be solved in this way, the operator will make a visit. The most common failures are caused because the main power source has been disconnected, the WiFi signal is weak for relocation of the router or a change in the WiFi password by owners.

On the other hand, the elimination of electronic components due to reaching their lifespan or failure due to inappropriate handling conditions will be collected and taken to confinements authorized by the health sector authorities.

The lifespan of the mechanical parts in the device is estimated up to ten years. The sensor’s lifespan depends to the manufacturer’s provisions. Considering the parts’ lifespan and cost of the electronic and mechanical components, it is appraised this system is profitable in the long term.

### 2.6. Characteristics of the Study Area

In Mexico, a rural community is one in which fewer than 2500 habitants live [39]. These provide food to the cities, since agriculture is principally developed there, in addition to some zones where aquaculture and fishing are the main sources of economic income. The plots of land where families live in the rural areas of Guasave, Sinaloa, are extensive, around 1600 m^2^, and some have a well to extract water. On these properties, backyard farming activities are performed that contribute to the income of the family economy and its food sustainability [40].

The water supply for domestic and backyard farming activities originated underground and is extracted through wells via electric pumps, since the communities of Guasave, Sinaloa, have electricity supplied by the Federal Electricity Commission (CFE), agency of the Mexican state. There are also Internet connectivity and wireless communications, so it is technically and economically possible to operate the IoT system after basic training is provided to members of the family where the system is installed.

Additionally, the municipality of Guasave has a wide network of interconnected roads and paths that give access to all communities within; this facilitates the maintenance of the devices.

The presence of the Pacific Ocean and the Sinaloa River in the study area are hydrological expressions that affect the level of groundwater in an unconfined coastal aquifer, combined with meteorological phenomena associated with prolonged periods of drought. Hurricanes that form in the Pacific Ocean and hit the Guasave Valley, Sinaloa, the intense pumping for agricultural irrigation, supply to aquaculture farms, backyard farming irrigation and domestic use mainly produce decreases in the piezometric level.

Given this scenario, the piezometric level must be monitored to avoid decreases in the level of continental water that could produce saline intrusion and thereby contaminate the aquifer with brackish water, unsuitable for agricultural activities, so a control of the piezometric level and the temperature of water are important, the latter being necessary data for water quality.

It is also important to control the level of groundwater to prevent it from dropping below the height of the well pumps or below the depth of the wells; if this happens, the communities would be left without water. The depth of the piezometric level in the Valley ranges between 1 and 19 m and is influenced by the recharge of the Sinaloa River. In the middle portion and close to the coastline, the level is less than 5 m deep [41] and maintains a fragile balance with sea level.

In the municipality of Guasave, there are 549 rural locations [42] where the IoT system can be installed, especially in properties with a well where the family lives, usually fenced properties, where vandalism is reduced due to these circumstances. The topographic relief of the valley is light, with a slope of 0.5 m/km, so communication via antennas is good.

### 2.7. Data Acquisition

On 23 June 2023, in the rural community of Agua Blanca, Guasave, Sinaloa, Mexico, the prototype was installed in a domestic well that has Internet connection via WiFi, compatible with the ESP8266 module. It also could be using a 4G modem connected to a public cellular network with a micro-Sim to provide the WiFi network with Internet access.

The elevation of the reference point (Zo) was obtained with differential GNSS in RTK mode (SimpleRTK2B-Ardusimple) that provides centimeter precision linked through the NTRIP protocol to a transmitter base (SimpleRTK2B-Ardusimple) georeferenced to the international reference frame (ITRF08).

At the time of the installation of the sensor, to calculate the total cable length that would be submerged for monitoring, we obtained a water depth of 1.95 m (d). Also, the pressure sensor range of measurement is 5 m of water column (φ); therefore, in this well and due to the shallow water conditions, it was considered to submerge 4 m of cable, that is, φ = 4 m and L = 5.95 m.

Table 1 shows the sensor installation data. The Identifier Name (Name ID), Elevation, Total Submerged Cable Length (L) and network credentials were added in situ to the ESP8266 module using WiFiManager. The position of the device, the elevation, and the hmin and hmax values were added directly to the server database for recording and comparing the piezometric level with the hmin and hmax values. If data sent by the sensor are lower than hmin, this means the water level has decreased below the sensor and this would not correspond to a valid measurement. Moreover, if the level sent by the sensor is above hmax, this means the water level has increased above the pressure sensor range of measurement of 5 m of water column (φ). For these cases, an alert was programmed indicating the Name ID sensor that requires attention. In the case of being below the hmin, it would be necessary to increase the total submerged cable length and, in the case of being higher than the hmax, the length of the submerged cable must be reduced at the operating threshold of the pressure sensor (5 m).

## 3. Results

### 3.1. Final View of the Prototype

The developed device is shown in Figure 7. Inside the pipe is the PCB and it has the shape of a reel to facilitate the winding of the 30 m cable. The device is powered by a 5 V 2 A micro-USB from an AC regulator.

**Figure 8:**
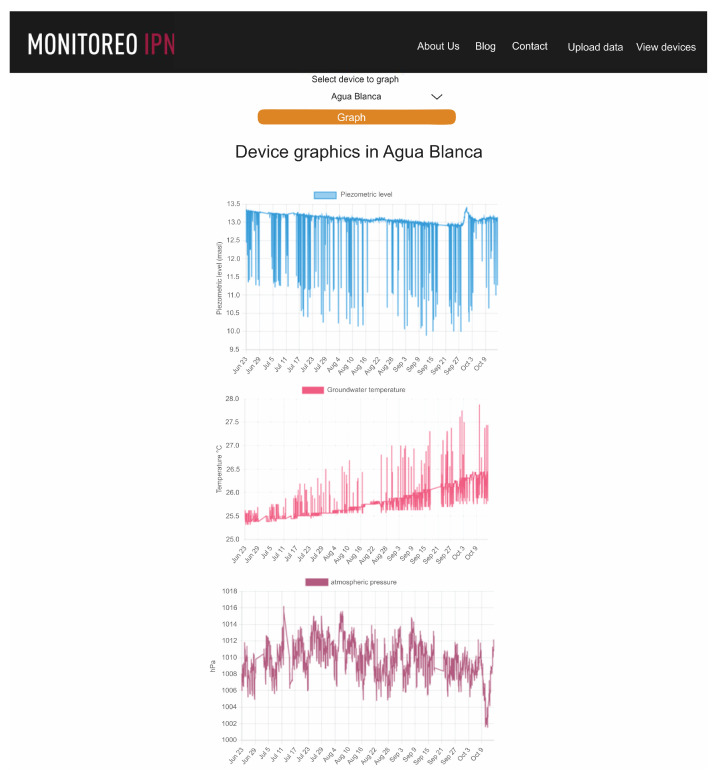
Raspberry pi server web page display.

Access to the data is achieved using a device with Internet access in any web browser with the link http://piezometriaguasave.ddns.net/aguablanca.php, accessed on 15 October 2023, connecting with the Raspberry web server. Figure 8 shows a visualization provided by the Raspberry pi web server of the information received by the prototype installed in the domestic well: piezometric level (upper), temperature (central) and atmospheric pressure (lower).

**Figure 9:**
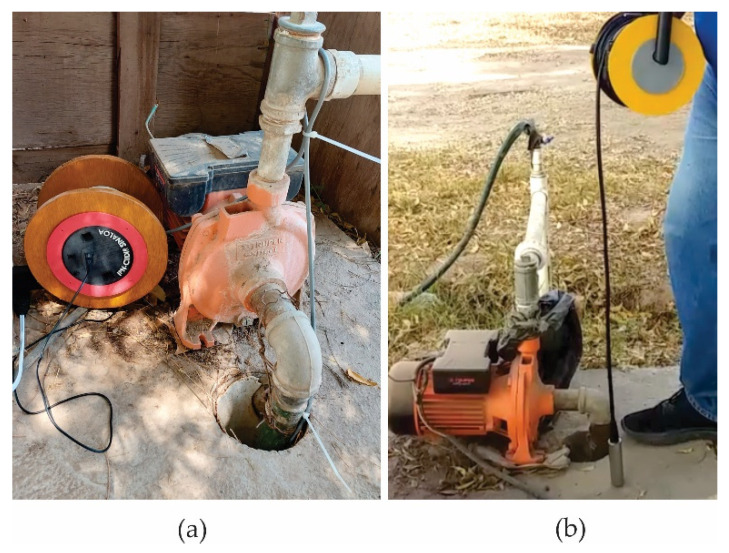
Domestic well under monitoring with identifying name “Agua Blanca” (**a**); control process at different depths for pressure sensor validation (**b**).

When inserting the probe with the sensor into the well, verification tests were run on site, so the device readings coincide with reality. This is estimated by measuring the depth of the water level with a manual probe and by introducing the pressure sensor at various controlled depths from the surface control point (Zo) (Figure 9b). In this way, it is verified that the measurement data from the server correspond with the direct measurement obtained through a vertical control of depth of the pressure sensor in the saturated zone of the well in Equation (2). In addition, once the measuring device is installed, the depth of the piezometric level in the well is randomly measured and this value is compared with the one recorded on the server, observing that they are the same values.

When the pumping starts, the levels drop in a shorter time, stabilizing afterwards, so to verify the operation of the device, the water level is measured before the pumping, then every five minutes, later on the sampling becomes more spaced considering the variation of the piezometric level. This way, the direct measurement is compared with the one recorded by the sensor, observing maximum differences no greater than 2 cm. Differences may be due to errors in the observation of measurements made with a probe or to the sensor response. These differences, whether due to an error in observation or variations in the precision or accuracy of the device, are within the practical tolerance threshold for the observed variable.

The temperature obtained with the device is validated by measurements on water samples taken on site, measured using a Hanna conductivity meter, model HI 98331. As temperature is a variable with minimum changes, the greatest gradient being the change from day to night, samples were taken every half hour for verification, with both measurements being consistent.

### 3.2. Data Analysis

Figure 8 shows the time series from 23 June to 14 October 2023; 49,713 data were downloaded from the Raspberry server corresponding to piezometric level, ambient temperature, groundwater temperature and atmospheric pressure. Sudden changes in the piezometric level are due to the water extraction pump operation. The trend of the curve is downward, meaning the piezometric level generally decreases as the summer progresses with a local upward change at the beginning of autumn. It is also clearly observed that for days in which there is no pumping or pumping ceases, the piezometric level is maintained. The diameter of the well is 4 inches, it has a depth of 12 m and the pump installed has a capacity of 2 L per second.

The temperature in the well water is practically stable, the average is 25.90 degrees Celsius with a standard deviation of 1.14 degrees Celsius. The temperature is validated with samples taken on site and measured with a Hanna model HI 98331 conductivity meter.

The ambient temperature is oscillating, with an arithmetic mean of 34.24 degrees Celsius and a standard deviation of 4.96 degrees Celsius and atmospheric pressure varies from 1006 to 1016 hPa.

### 3.3. Piezometric Level

**Figure 10:**
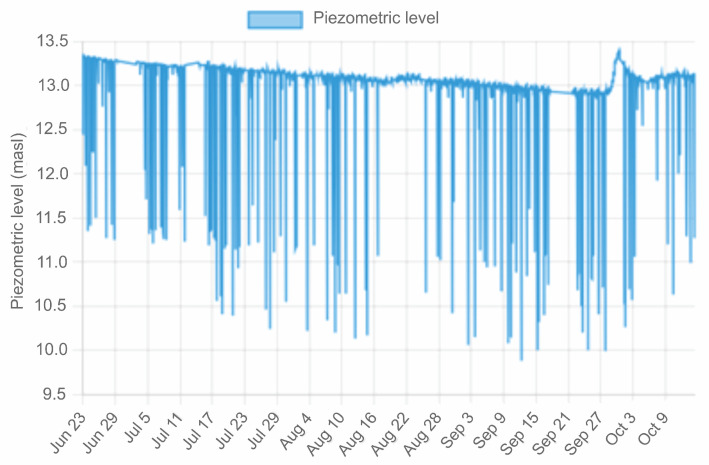
Graph of piezometric level records.

Figure 10 shows the behavior of the variation in the piezometric level of the “Agua blanca” site. Sudden drops and rises correspond to the pumping system operation. When the pump is turned on, the water table drops suddenly and then recovers in a short time, so this phenomenon is observed in the time series. During the recording period, in the process of recovery of the piezometric level, a downward trend of 0.5 m is observed in the recording period, which corresponds to the summer and early autumn period, which indicates that the aquifer did not recover to its levels, but they were depressed by the demand for water typical of the indicated seasonal period. The days when there is no pumping are also observed, even observing a slight rise in the piezometric level when pumping stops.

**Figure 11:**
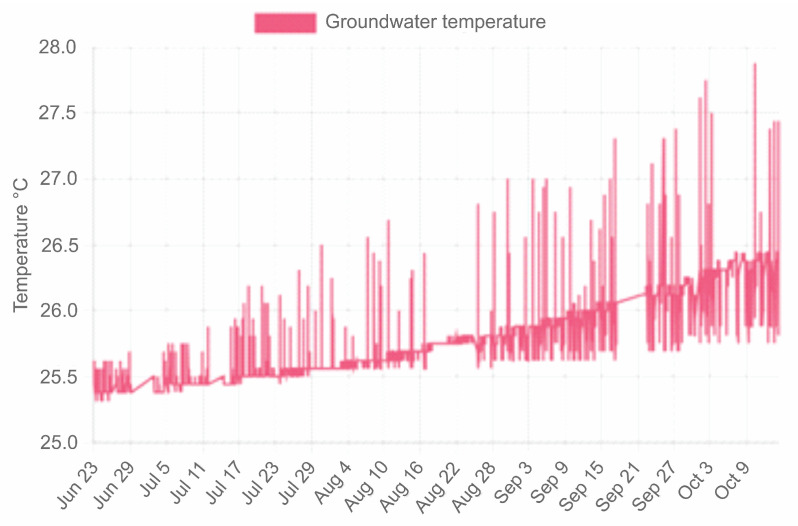
Groundwater temperature graph for sensor Ds18b20.

The groundwater temperature presented temporal regularity with a mean value of 25.90 degrees Celsius and a standard deviation of 1.14 °C (see Figure 11).

**Figure 12:**
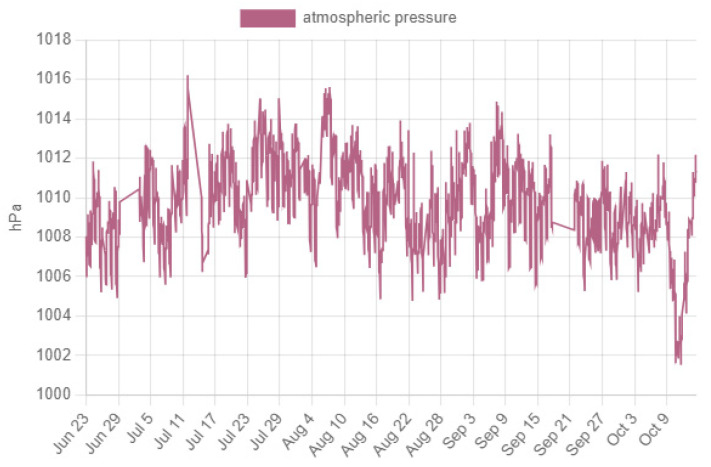
Atmospheric pressure graph, obtained with the BME280 sensor.

The atmospheric pressure presented values between 1006 hPa and 1015 hPa (Figure 12), with expected variations.

## 4. Discussion

Wireless sensors for measuring the piezometric level, such as the one described here, exhibit great importance in obtaining data in hydrology [28,42,43,44]. Those developed with IoT technology in groundwater management applications stand out in data acquisition, monitoring and information management [20,24].

In particular, low-cost wireless sensors are accessible to build due to the affordable cost of existing electronic and communication hardware for the generation of data in real time, the use of open-source software for data storage and visualization, the reduction in operational cost for installation and maintenance, the scalability and the volume of obtained measurements [11,22].

Other water level measurement prototypes have been developed with ultrasonic sensors [43], with pressure components (MS5803-14BA) encapsulated in a plastic container [44], and the recent pressure sensor with piezoresistive MEMS technology [45]. All these prototypes are in testing and operating stages; in addition, each one of them requires specific control calibration when installed. The most popular technique for measuring water level is commercial sensors (Solinst, HOBO, DIVER) that use pressure transducers [24].

Carderwood et al. [11] describes that a main component for continuous monitoring is found in the contribution of well owners. They observed interest in the owners for the availability of the data in a web interface. This component is presented as a strategy to facilitate the installation of sensors in existing wells. In this way, our prototype to measure the piezometric level of the aquifer in real time with a personal public web interface presents a low-cost scalability scenario. Furthermore, by combining GIS with the IoT system of this prototype, the quality of the generated database will improve [24].

## 5. Conclusions

A low-cost monitoring system was developed, designed to monitor in real time the piezometric level and temperature of water in wells, as well as atmospheric pressure and temperature. The sensor data are sent to a web page where the user can monitor the behavior of the levels in real time or store the data for later processes. The device was tested in a well, with reliable results, so it is possible to replicate it and place it in other wells to collect necessary data in hydrological studies, by configuring a network for monitoring groundwater and environmental variables such as atmospheric pressure and temperature.

The developed device is environmentally friendly and non-toxic. This system has an operating voltage of 5 V and a current consumption of 180 mA equivalent to a power of 0.9 W, so it has low electrical consumption. The developed system is transferable to any rural community that has electricity and Internet connectivity.

## Figures and Tables

**Table 1:** Sensor installation data in the domestic well.

Name ID	UTM Coordinates (Zone 12 N)		Zo (m)	L (m)	h min (m)	h max (m)
	X	Y				
Agua Blanca	746,991.62	2,831,081.19	15.85	5.95	9.9	14.9

## Data Availability

Data are contained within the article.
